# Prospective evaluation of a rapid diagnostic test for *Trypanosoma brucei gambiense* infection developed using recombinant antigens

**DOI:** 10.1371/journal.pntd.0006386

**Published:** 2018-03-28

**Authors:** Crispin Lumbala, Sylvain Biéler, Simon Kayembe, Jacquies Makabuza, Stefano Ongarello, Joseph Mathu Ndung’u

**Affiliations:** 1 Programme National de Lutte contre la Trypanosomiase Humaine Africaine, Kinshasa, Democratic Republic of the Congo; 2 Global Health Institute, University of Antwerp, Antwerp, Belgium; 3 Foundation for Innovative New Diagnostics (FIND), Geneva, Switzerland; Universiteit Antwerpen, BELGIUM

## Abstract

**Background:**

Diagnosis and treatment are central elements of strategies to control *Trypanosoma brucei gambiense* human African trypanosomiasis (HAT). Serological screening is a key entry point in diagnostic algorithms. The Card Agglutination Test for Trypanosomiasis (CATT) has been the most widely used screening test for decades, despite a number of practical limitations that were partially addressed by the introduction of rapid diagnostic tests (RDTs). However, current RDTs are manufactured using native antigens, which are challenging to produce.

**Methodology/Principal findings:**

The objective of this study was to evaluate the accuracy of a new RDT developed using recombinant antigens (SD BIOLINE HAT 2.0), in comparison with an RDT produced using native antigens (SD BIOLINE HAT) and CATT. A total of 57,632 individuals were screened in the Democratic Republic of the Congo, either passively at 10 health centres, or actively by 5 mobile teams, and 260 HAT cases were confirmed by parasitology. The highest sensitivity was achieved with the SD BIOLINE HAT 2.0 (71.2%), followed by CATT (62.5%) and the SD BIOLINE HAT (59.0%). The most specific test was CATT (99.2%), while the specificity of the SD BIOLINE HAT and SD BIOLINE HAT 2.0 were 98.9% and 98.1%, respectively. Sensitivity of the tests was lower than previously reported, as they identified cases from partially overlapping sub-populations. All three tests were significantly more sensitive in passive than in active screening. Combining two or three tests resulted in a markedly increased sensitivity: When the SD BIOLINE HAT was combined with the SD BIOLINE HAT 2.0, sensitivity reached 98.4% in passive and 83.0% in active screening.

**Conclusions/Significance:**

The recombinant antigen-based RDT was more sensitive than, and as specific as, the SD BIOLINE HAT. It was as sensitive as, but slightly less specific than CATT. While the practicality and cost-effectiveness of algorithms including several screening tests would need to be investigated, using two or more tests appears to enhance sensitivity of diagnostic algorithms, although some decrease in specificity is observed as well.

## Author summary

Sleeping sickness, or human African trypanosomiasis (HAT), is a neglected tropical disease that represents a risk to more than seventy million people in Sub-Saharan Africa. Most cases are caused by infection with *Trypanosoma brucei gambiense*. Diagnosis of HAT relies on the identification of suspected cases by serological methods, which include recently developed rapid diagnostic tests (RDTs). Current RDTs are produced using native antigens that are purified from live parasites in a laborious and dangerous process. The objective of this study was to evaluate the performance of a new RDT made using recombinant antigens, by screening people in fifteen endemic sites in the Democratic Republic of the Congo. The new RDT was found to be more sensitive than, and as specific as, the reference RDT made using native antigens. It was also more sensitive than CATT, a serological test that has been widely used for decades. While one third of HAT cases were correctly diagnosed by all tests, the other cases were only identified by one or two of the tests. In order to enhance case detection and accelerate elimination of HAT, there may be a need to explore diagnostic strategies that combine two or more screening tests.

## Introduction

Human African trypanosomiasis (HAT) is a vector-borne, neglected tropical disease, which puts 70 million people living in sub-Saharan African countries at risk [1]. The most common form of the disease is caused by infection with the protozoan parasite *Trypanosoma brucei gambiense* (g-HAT), which in 2015, accounted for more than 97% of all reported HAT cases [2]. Patients progress from an early disease stage that is characterized by the presence of trypanosomes in the blood and lymphatic system, to a late stage that is associated with the invasion of the central nervous system by parasites [3]. If left undiagnosed and untreated, the disease is generally fatal, although asymptomatic cases and others that progress spontaneously to apparently pathogen-free status have been reported [4].

Identification of serological suspects is the main entry point into diagnostic algorithms for g-HAT. The card agglutination test for trypanosomiasis (CATT/*T*.*b*. *gambiense*) has been the most commonly used screening test for g-HAT. It detects antibodies using a suspension of purified, fixed and stained bloodstream-form trypanosomes expressing LiTat 1.3 variant surface glycoprotein (VSG), a predominant variant antigen of *T*.*b*. *gambiense* [5]. While CATT has played a central role in the control of HAT, its large-scale implementation for passive screening in health facilities in remote locations has been limited due to operational challenges such as the need for an agitator, electricity and refrigeration. In some settings, the sensitivity and specificity of CATT have also been reported as being problematic [6].

In an effort to address the shortcomings of CATT, two rapid diagnostic tests (RDTs) that detect host antibodies have recently been developed, the HAT Sero-*K*-SeT manufactured by Coris BioConcept (Belgium), and the SD BIOLINE HAT, hereinafter referred to as “RDT1”, produced by Alere/Standard Diagnostics (SD, South Korea), which include the same two antigens, VSG LiTat 1.3 and VSG LiTat 1.5. These RDTs were evaluated in retrospective studies, with very promising performance results [7,8]. Evaluation of a prototype of the RDT1 in a prospective study in three endemic countries, Angola, the Democratic Republic of the Congo (DRC) and the Central African Republic, showed that the sensitivity of the RDT was not different from the sensitivity of CATT, while its specificity was 1.3% lower [9]. A prospective study using the HAT Sero-*K*-SeT also reported excellent performance [10]. A comparison of both RDTs in an independent study using stored plasma samples collected in Guinea and Côte d’Ivoire concluded that there was no difference in diagnostic accuracy between the two tests [11]. The RDTs have now been introduced in multiple HAT endemic countries, where they are being used in HAT elimination programmes. However, production of the native antigens used in the manufacture of the RDTs remains a challenge, as it relies on a labor-intensive, costly and risky process that involves inoculating rats with human-infective trypanosomes. To address this challenge, and to improve standardization and quality of manufacturing, a new RDT that is produced exclusively using recombinant antigens, the SD HAT BIOLINE 2.0 (“RDT2”), has been developed in a partnership facilitated by the Foundation for Innovative New Diagnostics (FIND).

The primary objective of this study was to evaluate the diagnostic accuracy of RDT2 in a multi-centric, prospective study in the DRC, and to demonstrate its non-inferiority to RDT1. As a secondary objective, the accuracy of RDT2 was compared to that of CATT.

## Methods

### Enrolment of participants

Study participants were enrolled from 6 June 2015 to 5 January 2016 in the Bandundu Province of the DRC by passive screening in ten health facilities, and by active screening using five mobile teams of the Programme National de Lutte contre la Trypanosomiase Humaine Africaine (PNLTHA) of the DRC (Table 1). In the health facilities, participants were enrolled among patients presenting themselves or referred from other health facilities after suspicion of HAT, and among relatives who accompanied patients. During active screening, anybody who presented to the mobile team was eligible for enrolment in the study. Study sites were visited by an external monitor prior to commencement of the study to verify that they were adequately prepared and personnel properly trained, and during the study to verify that the protocol was being adhered to. HAT cases were defined as subjects in whom trypanosomes were demonstrated by microscopy in either lymph node aspirate, blood or cerebrospinal fluid (CSF). All positive parasitology results were verified by the site supervisor. Cases were classified as early stage when no trypanosomes were observed in their CSF, and the CSF white cell count was lower than or equal to 5 cells/μL, while those with trypanosomes in the CSF and/or a cell count above 5 cells/μL were classified as late stage [12]. Controls were subjects living in the same areas as cases, with no known history of HAT infection, and who were either negative with all three screening tests, or who were positive with one or several screening tests, but in whom no parasites were detected in any body fluid. Clinical signs and symptoms were not considered exclusion criteria for controls.

**Table 1:** Study sites and the corresponding numbers of HAT cases and controls that were enrolled.

Site name	Site type	HAT cases	Controls
Nkara	Fixed facility (HS)	41	1,698
Masi-Manimba	Fixed facility (HGR)	31	1,521
Masamuna	Fixed facility (CS)	18	2,041
Kwamouth	Fixed facility (HGR)	8	784
Kitoy	Fixed facility (HS)	8	951
Bagata	Fixed facility (HGR)	5	420
Yasa Bonga	Fixed facility (HGR)	5	761
Bangumi	Fixed facility (CS)	3	321
Bandundu	Fixed facility (CDTC)	3	1,184
Bandundu	Fixed facility (HGR)	0	934
Kwamouth	Mobile team	45	7,326
Mushie	Mobile team	39	7,279
Bandundu	Mobile team	20	19,457
Idiofa	Mobile team	21	7,737
Mokala	Mobile team	13	3,855
*All sites*		*260*	*56*,*269*

HS: "Hôpital Secondaire"; HGR: “Hôpital Général de Référence”; CS: “Centre de Santé”; CDTC: “Centre de Dépistage, Traitement et Contrôle”.

### Tests performed

The RDT2 (SD, South Korea) is an immuno-chromatographic test for qualitative detection of antibodies of all isotypes (IgG, IgA and IgM). It includes a nitrocellulose membrane strip with two test regions (T1 and T2) that are pre-coated with two recombinant antigens. T1 is coated with Invariant Surface Glycoprotein 65–1 (ISG65) expressed in *Escherichia coli* [13] and T2 with the N-terminal domain of Variant Surface Glycoprotein LiTat 1.5 (VSG LiTat 1.5) produced using a Baculovirus expression system. A procedural control line (C) is also included. The test is stable for at least 24 months at 40°C, or 5 weeks at 55°C. The test is performed in the same way as RDT1, as described by Lumbala et al. [14]. In summary, a sample of 20 μl of whole blood is taken from a finger prick and transferred into a sample well using a disposable plastic capillary tube, and 4 drops (approximately 120 μl) of test diluent are then added. The sample flows along the membrane by capillarity, passing through the test regions T1 and T2. Results are read after 15 to 20 minutes by comparing the intensity of the test lines against a colour chart provided by the manufacturer. A result is considered positive when the control line C and either one or both T1 and T2 test lines are visible (regardless of their intensity), negative when only the C line is observed, and invalid if the C line is not observed. In active screening, all participants found positive with a HAT screening test were also tested for malaria using an RDT (SD BIOLINE Ag P.f), while in passive screening, all participants were tested with a malaria RDT (S3 Table). However, results of malaria RDTs were only recorded for subjects who were eligible for enrolment (see below). Those who tested positive for malaria were examined, and if necessary, treated in line with national guidelines.

Three screening tests (CATT, RDT1 and RDT2) were performed on finger-prick blood from each subject who presented to mobile teams, any subject who presented to a health facility with symptoms indicative of HAT, and accompanying individuals who consented to participate in the study. The results of screening tests were read by two independent laboratory technicians or nurses, and the results recorded separately. To avoid overburdening study teams and to keep the study design as simple as possible, CATT was only performed on whole blood, and not on diluted plasma. Similarly, the trypanolysis test was not performed during this study, as this would have required additional resources to collect and transport samples for analysis, which at that time could not be performed in DRC.

In both active and passive enrolment, any subject who was positive with at least one of the screening tests, or who showed symptoms highly suggestive of HAT, was eligible for immediate enrolment in the subsequent parasitological work-up. Written informed consent was sought from these subjects prior to enrolment. Any individual who declined to participate in the study was managed according to the standard procedures of the PNLTHA. Individuals who were negative to all three screening tests and who had no symptoms highly suggestive of HAT were not investigated further. Persons with palpable cervical lymph nodes had a lymph node aspirate taken and examined for motile parasites by bright field microscopy. A sample of 5 ml of venous blood was collected from each participant in a heparinized tube. Three hundred μL of blood was used to perform the capillary tube centrifugation (CTC) test (4 capillary tubes of approximately 65–70 μL) [15]. If the result of CTC was negative, 500 μL of whole blood was used to perform the mini anion exchange centrifugation technique (mAECT-wb) [16] and the remaining volume of blood (4.2 ml) was centrifuged to perform mAECT on buffy coat (mAECT-bc) as described by Camara et al. [17]. Since the mAECT-bc procedure had only been evaluated in one study in DRC, we took advantage of this study to collect some additional performance data to compare it with mAECT-wb, even though mAECT was only performed on a subset of cases. A lumbar puncture was performed on all HAT cases confirmed by any of the parasitological methods, as well as on other participants with clinical signs that were strongly suggestive of HAT, according to routine procedures. Parasitological examination of CSF was done using the modified single centrifugation technique [16]. The technicians who performed the tests were employees of the PNLTHA, with experience in performing routine parasitological tests for detection of trypanosomes. Training of personnel of mobile teams and fixed health facilities included how to perform, read and interpret results of the RDTs, the study protocol and related SOPs, completion of CRFs and data management. Any positive or doubtful parasitology result was verified and confirmed by the site supervisor. Participants with any missing screening test or parasitology results were excluded from the study. All the HAT cases that were identified during the study were treated according to national guidelines.

Two levels of blinding were adopted. During the initial screening of participants using blood from a finger prick, three health workers were each responsible for performing one of the three screening tests. The health workers operated independently (but used blood from the same finger prick), without exchanging results (first level of blinding), and did not have access to any clinical information. A supervisor was responsible for collecting results of the tests and deciding whether or not to collect venous blood for parasitological tests. Samples of venous blood were labelled with blinding codes by the supervisor (second level of blinding). The same codes were used to identify all samples collected from the participants (i.e. blood, lymph node aspirate, CSF) and constituted the anonymisation process that was maintained throughout the entire study.

### Data management and statistical analysis

Participant information and test results were recorded at study sites on paper case report forms, which were transferred to PNLTHA in Kinshasa for double data entry using a web-based clinical data management platform (VisionForm). Since two independent readings were available for each test and each sample, an approach based on bootstrapping resampling [18] was adopted: At each iteration, a random sequence of readings from the available data was generated (one reading per patient and per test) and used to calculate the performance metrics. This process was repeated (2,000 iterations per metric) to generate an empirical distribution of values for each metric, from which it was possible to derive values for the sample mean and 95% confidence intervals as bootstrapped percentiles.

Estimates of sensitivity and specificity were calculated for each screening test, on the overall data, and stratified by disease stage and by screening method (i.e. active and passive screening). The diagnostic performance of each antigen in the RDTs was also calculated. Sensitivity and specificity were defined as the percentage of HAT cases that were found positive and the percentage of controls that were found negative, respectively. Accuracy was assessed by calculating Youden’s index [19]. To evaluate the agreement between readers, Cohen’s Kappa factor was calculated. The statistical analysis was performed in the R statistical environment (version 3.2.3).

### Sample size calculation

The sample size was calculated to demonstrate non-inferiority of the sensitivity and specificity of RDT2 in comparison to RDT1. Based on the sensitivity of RDT1 of 92.0% that was reported by Lumbala et al. [14], using a non-inferiority margin of 8%, a confidence level of 5% and a power of 80%, the required number of HAT cases was calculated to be at least 143. Based on the same report, the expected specificity of RDT1 was 97.1%. Using a non-inferiority margin of 1%, a confidence level of 5% and a power of 90%, it was calculated that a minimum of 4,775 controls would be needed [20]. Based on the expected prevalence of HAT in the study area, the minimum number of subjects estimated to be screened in order to enrol 143 cases was 44,700.

### Ethical considerations

The study received ethical clearance from the School of Public Health of the University of Kinshasa (authorization number ESP/CE/012/2015). Participants provided written informed consent before being enrolled in the study. For children below 18 years, consent was provided by a parent or guardian. All individuals who presented at study sites during the period of enrolment and consented to being screened were eligible. Those who presented for screening but did not wish to participate in the study were screened according to the procedures of the PNLTHA. All participants’ samples were blinded and further analysed anonymously.

## Results

**Fig 1:**
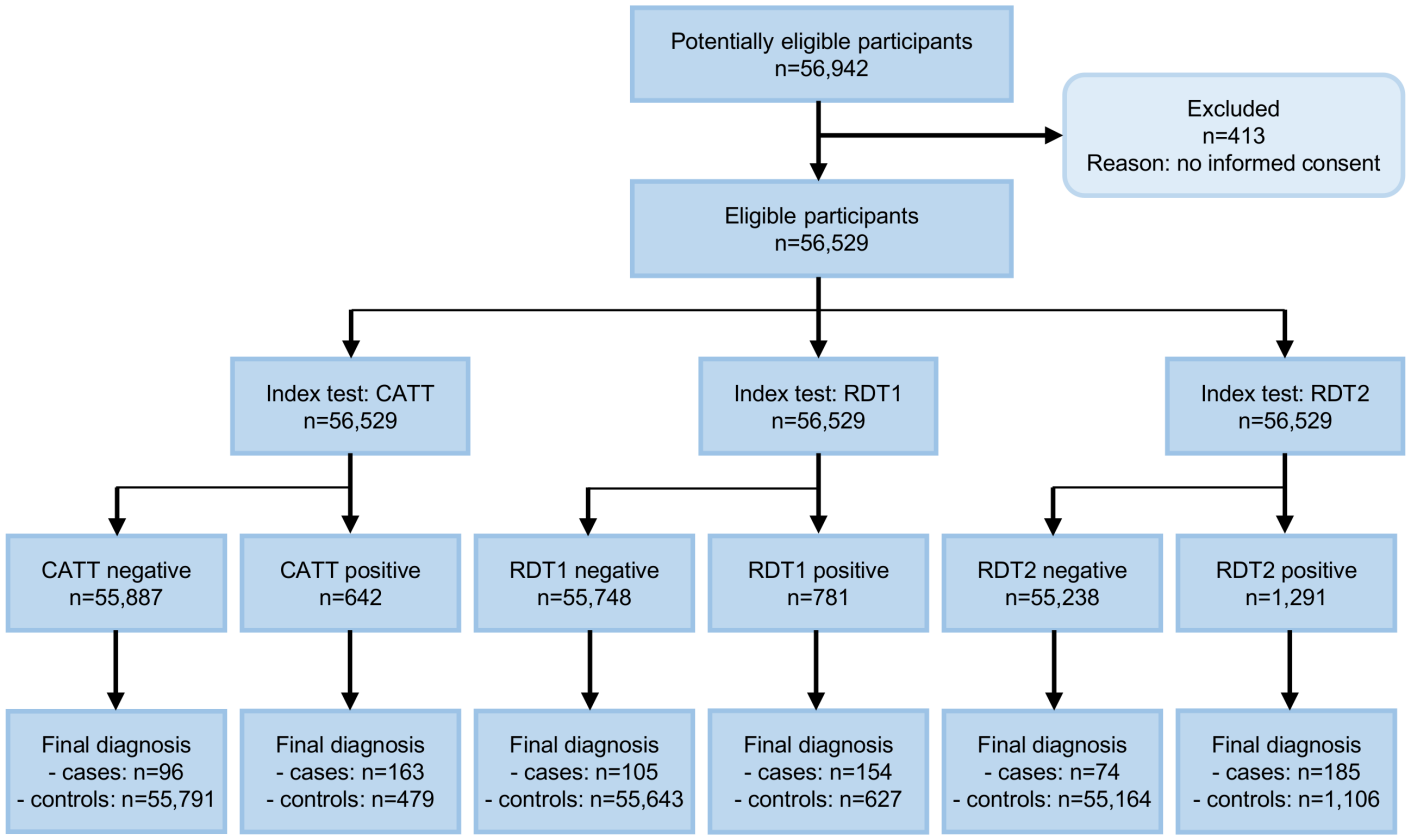
STARD diagram describing the flow of participants through the study.

A total of 260 HAT cases and 56,269 controls were enrolled after screening 56,942 people. 413 individuals could not be included in the study because they did not provide informed consent (Fig 1). A total of 138 (53%) cases and 45,654 controls were enrolled by active screening, while 122 (47%) cases and 10,615 controls were enrolled by passive screening. Among cases, the early stage to late stage ratio was 4.3 in active screening and 0.53 in passive screening. The HAT prevalence was 0.30% in active and 1.13% in passive screening. On average, 255 persons were tested per day by each mobile team.

**Fig 2:**
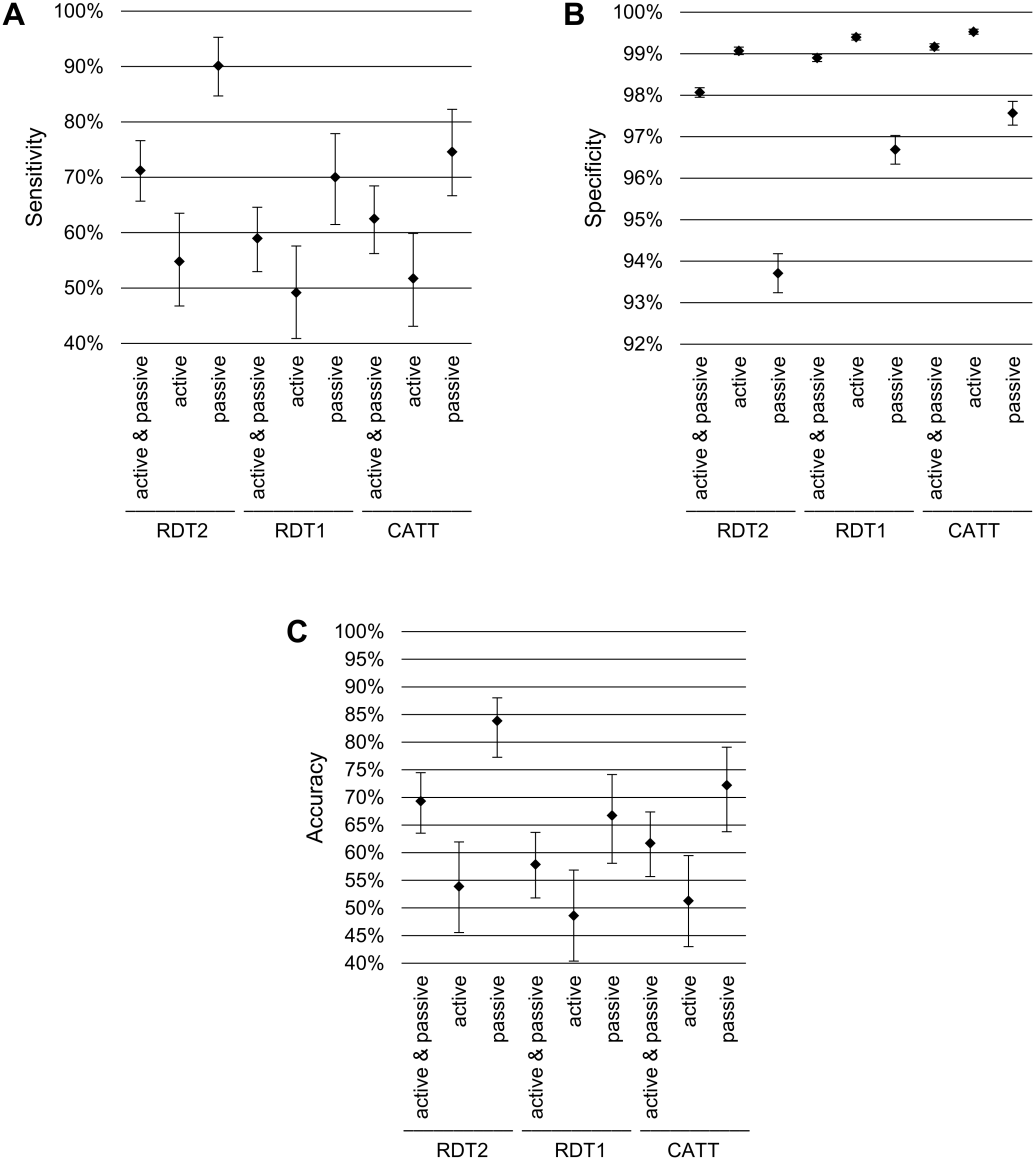
Sensitivity (A), specificity (B) and accuracy (C) of the RDT2, RDT1 and CATT tests, by screening method. RDT1: SD BIOLINE HAT rapid diagnostic test; RDT2: SD BIOLINE HAT 2.0 rapid diagnostic test; CATT: card agglutination test for trypanosomiasis.

The estimates of sensitivity, specificity and accuracy of the RDT2, RDT1 and CATT tests in active screening, passive screening, and active and passive screening combined are shown in Fig 2. When the results of active and passive screening were combined, the sensitivity of the three screening tests was unexpectedly low. While RDT2 detected 71.2% [CI: 65.7%; 76.6%] of the HAT cases, CATT detected only 62.5% [CI: 56.2%; 68.4%] and RDT1 only 59.0% [CI: 53.0%; 64.6%] of the cases. Sensitivity was particularly low in active screening, with only 54.8% [CI: 46.8%; 63.5%], 51.8% [CI: 43.1%; 59.9%] and 49.2% [CI: 40.9%; 57.6%] of cases being detected by the RDT2, CATT and RDT1, respectively. In passive screening, the three tests were more sensitive, with RDT2 achieving the highest sensitivity (90.1% [CI: 84.7%; 95.3%]), followed by CATT (74.6% [CI: 66.7%; 82.3%]) and RDT1 (70.0% [CI: 61.5%; 77.9%]).

CATT had the best specificity (99.2% [CI: 99.1%; 99.2%]), followed closely by RDT1 (98.9% [CI: 98.8%; 99.0%]) and RDT2 (98.1% [CI: 98.0%; 98.2%]). With all the screening tests, specificity was significantly higher in active than in passive screening. In active screening, specificity was highest with CATT (99.5% [CI: 99.5%; 99.6%]), which was followed by RDT1 (99.4% [CI: 99.3%; 99.5%]) and RDT2 (99.1% [99.0%; 99.2%]). Similarly, in passive screening, specificity was highest with CATT (97.6% [CI: 97.3%; 97.9%]), while lower results were obtained with RDT1 (96.7% [CI: 96.3%; 97.0%]) and RDT2 (93.7% [CI: 93.2%; 94.2%]).

RDT2 had the highest accuracy (69.3% [CI: 63.5%; 74.5%]), followed by CATT (61.7% [CI: 55.7%; 67.4%]) and RDT1 (57.9% [CI: 51.8%-63.7%]). All tests had a higher accuracy in passive than in active screening.

The agreement between the two technicians who read the screening tests was excellent. Cohen’s Kappa factor was above 99.8% with all the tests, both in active and passive screening.

The differences in sensitivity and specificity between two screening tests are shown in Table 2 for each possible pair of tests. The RDT2 was 12.3% [CI: 3.8%; 20.7%] more sensitive than the RDT1 when the results of active and passive screening were considered together. The difference was particularly pronounced in passive screening, where the sensitivity of RDT2 was 20.1% [CI: 9.4%; 29.8%] higher than that of RDT1. By contrast, there was no evidence of a difference in sensitivity between RDT1 and RDT2 in active screening (+5.6% [CI: -7.4%; 18.7%]). The objective of non-inferiority using a margin of 8% was met in both active and passive screening. The RDT2 was also more sensitive than CATT (+8.7% [CI: 1.0%; 16.6%]) when the results of active and passive screening were combined, and again, this effect was stronger in passive than in active screening (+15.5% [CI: 7.5%; 24.3%]). There was no evidence of a difference in sensitivity between RDT1 and CATT in both active (-2.6% [CI: -15.2%; 9.5%]) and passive screening (-4.6% [CI: -13.3%; 5.0%]).

**Table 2:** Differences in sensitivity and specificity between screening tests, and by method of screening.

Tests compared	Screening method	Difference (%)	
		Sensitivity (95% CI)	Specificity (95% CI)
RDT2−RDT1*	Both active and passive	12.3 (3.8;20.7)^#^	-0.83 (-0.96;-0.70) ^#^
	Active	5.6 (-7.4;18.7)	-0.33 (-0.44;-0.23) ^#^
	Passive	20.1 (9.4;29.8) ^#^	-2.98 (-3.50;-2.48) ^#^
RDT2−CATT*	Both active and passive	8.7 (1.0;16.6) ^#^	-1.10 (-1.22;-0.98) ^#^
	Active	3.1 (-9.8;15.5)	-0.46 (-0.56;-0.37) ^#^
	Passive	15.5 (7.5;24.3) ^#^	-3.86 (-4.37;-3.39) ^#^
RDT1−CATT*	Both active and passive	-3.6 (-11.3;4.4)	-0.27 (-0.37;-0.17) ^#^
	Active	-2.6 (-15.2;9.5)	-0.13 (-0.21;-0.05) ^#^
	Passive	-4.6 (-13.3;5.0)	-0.88 (-1.28;-0.50) ^#^

RDT1: SD BIOLINE HAT rapid diagnostic test; RDT2: SD BIOLINE HAT 2.0 rapid diagnostic test; CATT: card agglutination test for trypanosomiasis.

^#^ Difference that is significant at the 5% level.

* “RDT2−RDT1” corresponds to the performance of RDT2 minus the performance of RDT1, and likewise for the other pairs of tests.

The RDT2 was 0.83% less specific [CI: -0.96%; -0.70%] than RDT1 when results of active and passive screening were combined, which was within the non-inferiority margin of 1%. While the difference in specificity was minimal in active screening (-0.33% [CI: -0.44%; -0.23%]), it was more pronounced in passive screening (-2.98% [CI: -3.50%; -2.48%]). The RDT2 was also less specific than CATT (-1.10% [CI: -1.22%; -0.98%]), and this difference was more pronounced in passive (-3.86% [CI: -4.37%; -3.39%]) than in active screening (-0.46% [CI: -0.56%; -0.37%]). The RDT1 was slightly less specific than CATT (-0.27% [CI: -0.37%; -0.17%]), and this difference was also more pronounced in passive (-0.88% [CI: -1.28%; -0.50%]) than in active screening (-0.13% [CI: -0.21%; -0.05%]).

Considering that RDT1 and RDT2 are each made using two different antigens, we calculated the sensitivity and specificity of individual antigens. Table 3 shows that for each RDT, individual antigens detected partially overlapping groups of HAT cases, since the sensitivity obtained with single antigens was lower than the result of the RDT. Therefore, having two antigens in these tests resulted in higher sensitivity than if only one antigen had been used. While each of the antigens in RDT2 detected almost the same number of cases and contributed almost equally to the sensitivity of this test, one of the antigens of RDT1 (native VSG LiTat 1.3) detected a larger number of cases than the other antigen (native VSG LiTat 1.5). Similarly, Table 3 shows that the individual antigens of RDT2 contributed almost equally to specificity, while in the case of RDT1, native VSG LiTat 1.3 gave a slightly greater number of false positive results than the other antigen. All antigens were significantly more sensitive in passive than in active screening. The strongest difference was observed with recombinant ISG65 and recombinant VSG LiTat 1.5, whose sensitivity was two times higher in passive than in active screening.

**Table 3:** Sensitivity and specificity of individual antigens in screening tests, by screening method.

Antigen	Screening method	Sensitivity (95% CI)	Specificity (95% CI)
Recombinant ISG65	Both active and passive	55.0 (49.0;60.9)	98.9 (98.8;99.0)
	Active	37.4 (29.2;45.7)	99.5 (99.4;99.5)
	Passive	74.7 (65.0;82.3)	96.6 (96.3;97.0)
Recombinant VSG LiTat 1.5	Both active and passive	55.3 (49.2;61.3)	98.8 (98.7;98.9)
	Active	37.4 (29.3;45.5)	99.4 (99.4;99.5)
	Passive	75.4 (67.3;82.7)	96.1 (95.7;96.4)
Native VSG LiTat 1.3	Both active and passive	52.7 (46.7;58.6)	99.1 (99.0;99.2)
	Active	40.2 (31.7;48.3)	99.5 (99.5;99.6)
	Passive	66.8 (59.0;74.0)	97.3 (97.1;97.6)
Native VSG LiTat 1.5	Both active and passive	47.0 (40.8;52.8)	99.3 (99.3;99.4)
	Active	38.8 (30.6;47.2)	99.6 (99.5;99.7)
	Passive	56.4 (47.2;65.8)	98.2 (98.0;98.5)
CATT antigen (trypanosomes expressing VSG LiTat 1.3)	Both active and passive	62.5 (56.2;68.4)	99.2 (99.1;99.2)
	Active	51.8 (43.1;59.9)	99.5 (99.5;99.6)
	Passive	74.6 (66.7;82.3)	97.6 (97.3;97.9)

The three screening tests were significantly more sensitive in late stage than in early stage patients, as shown in Table 4. The strongest difference in sensitivity between stages was observed with CATT, whose sensitivity went up from 50.6% [CI: 42.7%; 58.8%] in early stage patients to 80.2% [CI: 72.3%; 87.8] in late stage patients. The RDT2 was the most sensitive in both early stage (59.8% [CI: 52.2%; 67.3%]) and late stage patients (87.9% [81.3%; 93.7%]). Similarly, all the individual RDT antigens were significantly more sensitive in late than in early stage patients. The largest difference between stages was observed with recombinant VSG LiTat 1.5 (42.1%), while the smallest difference was with native VSG LiTat 1.3 (27.0%). The most sensitive antigen in early stage patients was native VSG LiTat 1.3 (41.9% [34.0%; 49.4%]), while the most sensitive antigen in late stage patients was recombinant VSG LiTat 1.5 (80.4% [72.7%; 87.8%]).

**Table 4:** Sensitivity of the three screening tests and individual antigens, by disease stage.

Test	Antigen (s)	Disease stage	Sensitivity (95% CI)
RDT2	Recombinant ISG65 and recombinant VSG LiTat 1.5	Early	59.8 (52.2;67.3)
		Late	87.9 (81.3;93.7)
	Recombinant ISG65	Early	40.5 (32.6;47.9)
		Late	76.6 (67.9;84.5)
	Recombinant VSG LiTat 1.5	Early	38.2 (30.7;45.9)
		Late	80.4 (72.7;87.8)
RDT1	Native VSG LiTat 1.3 and native VSG LiTat 1.5	Early	49.7 (41.9;57.8)
		Late	73.2 (64.4;81.7)
	Native VSG LiTat 1.3	Early	41.9 (34.0;49.4)
		Late	68.9 (60.2;77.5)
	Native VSG LiTat 1.5	Early	34.6 (27.2;42.4)
		Late	65.7 (56.5;74.5)
CATT	Trypanosomes expressing VSG LiTat 1.3	Early	50.6 (42.7;58.8)
		Late	80.2 (72.3;87.8)

RDT1: SD BIOLINE HAT rapid diagnostic test; RDT2: SD BIOLINE HAT 2.0 rapid diagnostic test; CATT: card agglutination test for trypanosomiasis; VSG: variant surface glycoprotein.

**Fig 3:**
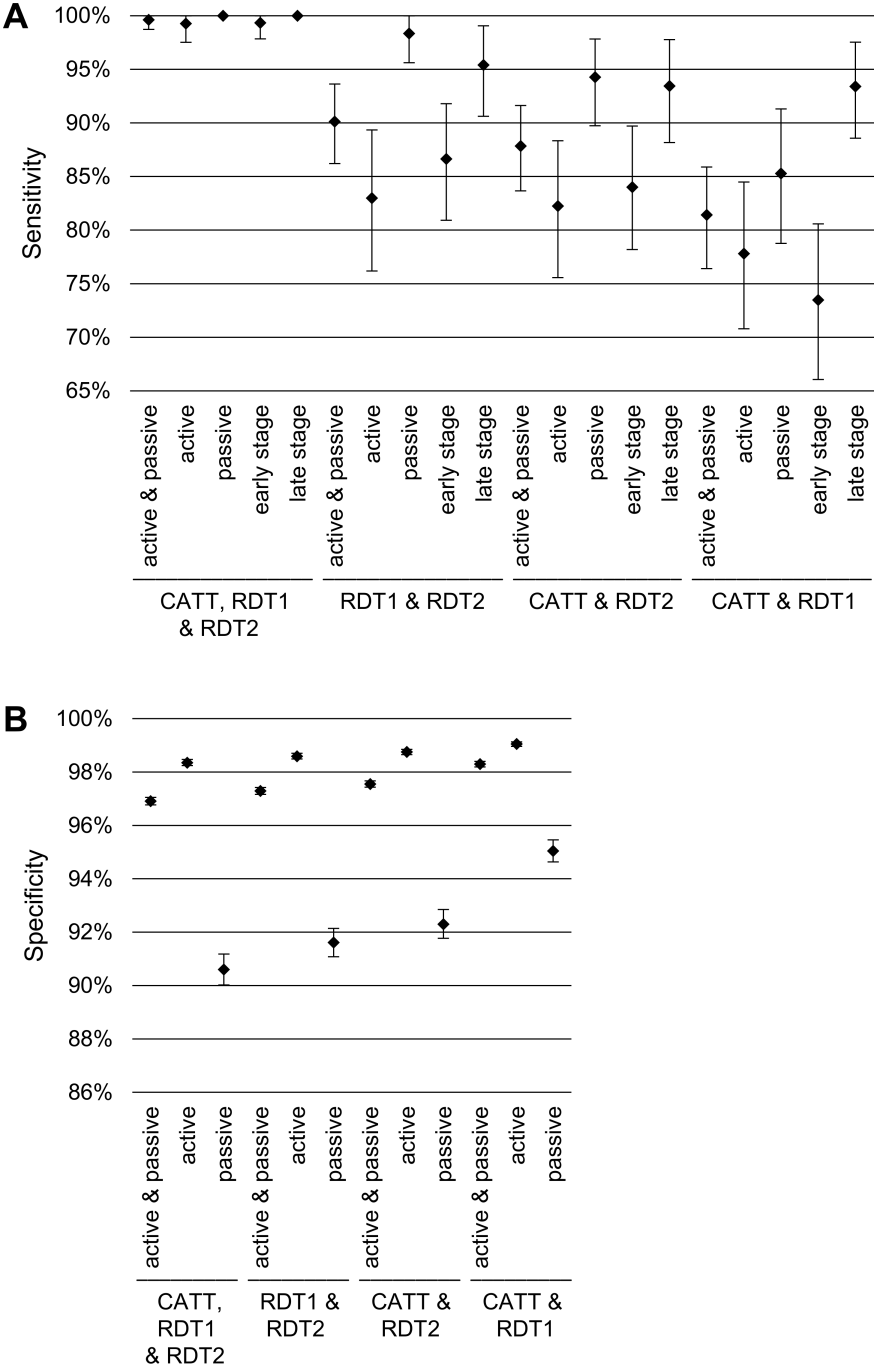
Sensitivity (A) and specificity (B) of all possible combinations of two or three screening tests, by screening method and by disease stage. Test combinations are shown in descending order of sensitivity. RDT1: SD BIOLINE HAT rapid diagnostic test; RDT2: SD BIOLINE HAT 2.0 rapid diagnostic test; CATT: card agglutination test for trypanosomiasis. The result of the combination of tests is positive if at least one of the tests is positive, while the result is negative if all the tests of the combination are negative.

We also calculated the diagnostic performance that would be achieved by combining two or three screening tests, with the goal of improving the overall sensitivity of screening, which is important in enhancing control of HAT, as humans are considered the main reservoirs of the disease [21]. The sensitivity and specificity of all possible combinations of two or three screening tests is shown in Fig 3. As expected, the highest sensitivity was achieved by combining all three tests (99.6% [CI: 98.7; 100.0]). This did not reach 100% because there were some differences between the two readers who interpreted test results. The most sensitive combination of two tests was RDT1 and RDT2, which detected 90.1% of cases [CI: 86.2%; 93.6%] and was markedly more sensitive than the individual tests. Lower sensitivity values were obtained by combining CATT and RDT2 (87.8% [CI: 83.7%; 91.6%]) and even more so by combining CATT and RDT1 (81.4% [CI: 76.4; 85.9]). Combining screening tests provided a greater increase in sensitivity in active than in passive screening. In active screening, sensitivity increased from 54.8% [CI: 46.8%; 63.5] with RDT2 to 83.0% [CI: 76.2%; 89.3%] when combining RDT1 and RDT2. In passive screening, this same combination achieved a remarkable sensitivity of 98.4% [CI: 95.6%; 100.0%], compared to 90.1% [CI: 84.7%; 95.3%] with RDT2 alone. In other words, combining these two RDTs would mean that only 1.6% of cases would have been missed in passive screening, while 9.9% of them would have remained undiagnosed using RDT2 only. However, using such combinations resulted in some trade-off in specificity, which went down to 96.9% [CI: 96.8%; 97.1%] when the three tests were taken together, or to 97.3% [CI: 97.2%; 97.4%] when combining RDT1 and RDT2.

**Fig 4:**
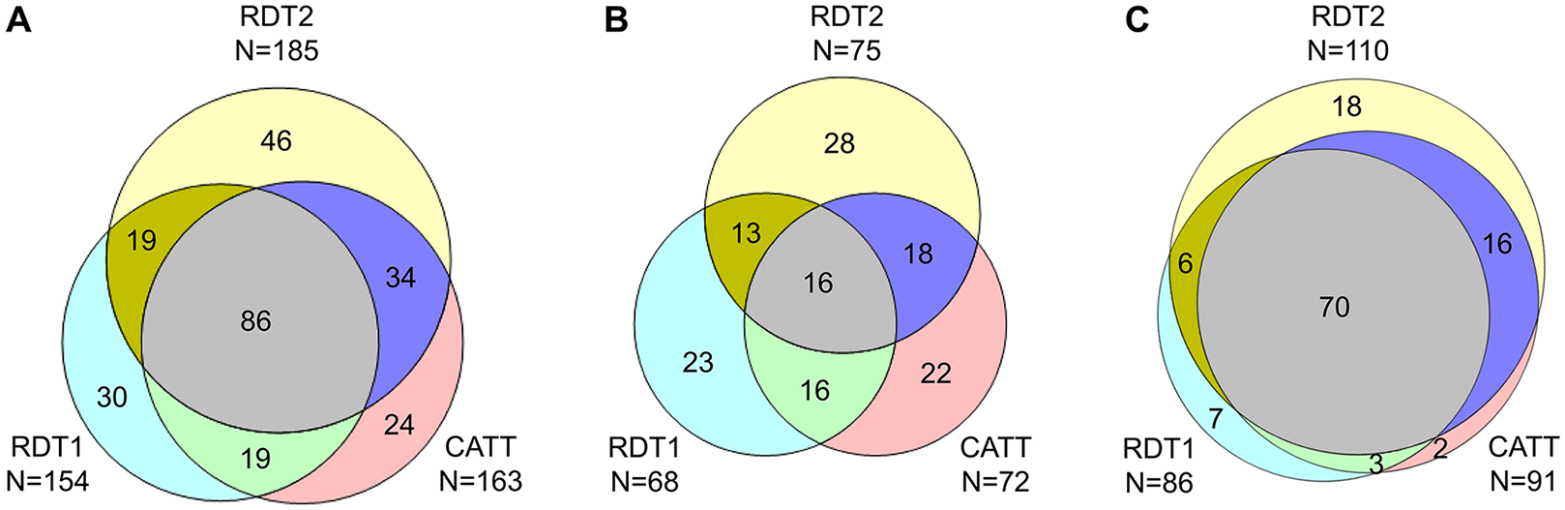
Venn diagrams showing the number of true positive results obtained with the RDT2, RDT1 and CATT tests. (A) Results from active and passive screening combined (N = 258 true positives); (B) results from active screening (N = 136 true positives); (C) results from passive screening (N = 122 true positives). For the sake of simplicity, only results obtained by the first reader are shown. The total number of true positives does not equal the total number of cases enrolled in the study (N = 260), as the first reader missed two cases in active screening.

**Fig 5:**
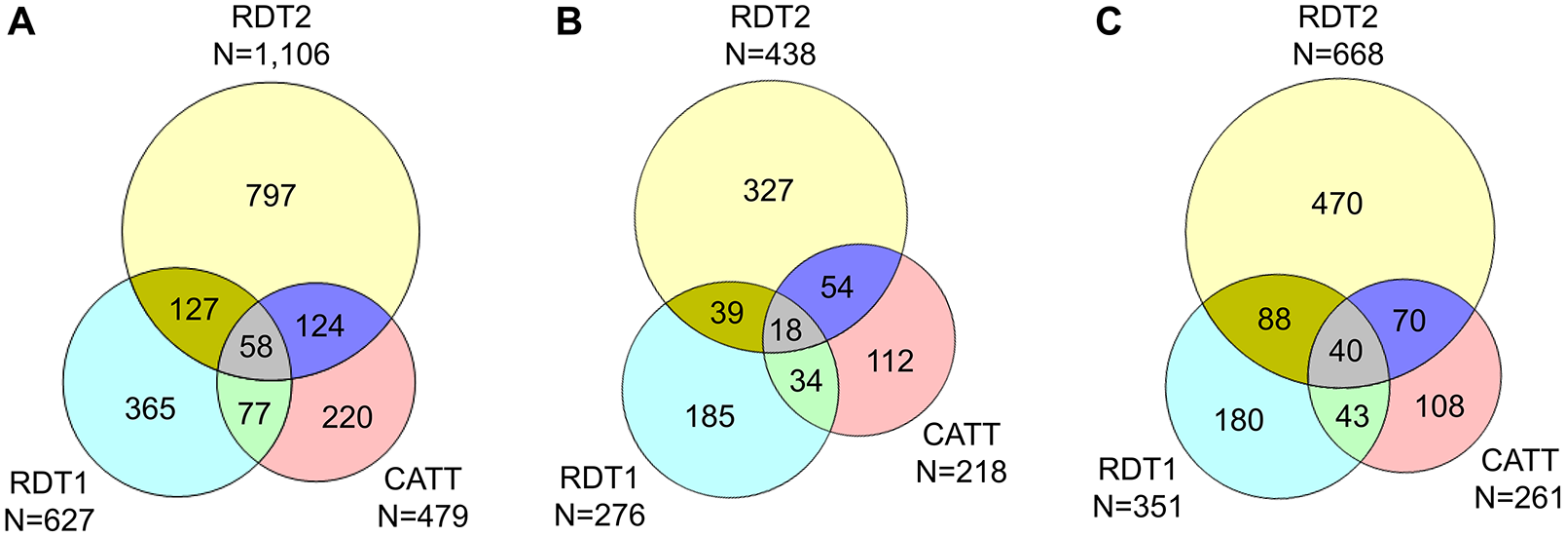
Venn diagrams showing the number of false positive results obtained with the RDT2, RDT1 and CATT tests. (A) Results from active and passive screening combined (N = 1,768 false positives); (B) results from active screening (N = 769 false positives); (C) results from passive screening (N = 999 false positives). For the sake of simplicity, only results obtained by the first reader are shown.

The contribution of each screening test to the detection of cases and to false positive results is demonstrated using Venn diagrams in Figs 4 and 5. Fig 4 shows that for true positive results, the degree of overlap between the tests was much higher in passive than in active screening. Fig 5 shows that for false positive results, the degree of overlap between the tests was also higher in passive than in active screening, but this difference was much less pronounced than for true positive results.

Both mAECT tests were performed on 124 HAT cases. Ninety cases (72.6%) were positive by mAECT-bc, while only 64 cases (51.6%) were positive by mAECT-wb. There was a high degree of overlap between the two tests, with 58 cases detected using both methods.

The number of HAT cases identified using the different parasitological tests used in the study, as well as the corresponding positivity rates of each of the screening tests, are included as supporting information (S2 Table). This data indicates that among the three parasitological tests performed on blood samples, the positivity rates of screening tests were highest in cases identified by mAECT-bc and lowest in cases diagnosed by CTC. The positivity rates of screening tests in cases identified by examining lymph node aspirates were not significantly different from the positivity rates obtained in cases that were positive with parasitological tests performed on blood samples. The highest screening test positivity rates were obtained in cases with trypanosomes detected in the cerebrospinal fluid.

## Discussion

The main objective of this study, to demonstrate the non-inferiority of the sensitivity and specificity of the RDT2 in comparison to the RDT1, was successfully achieved. However, all three screening tests that were evaluated were unexpectedly insensitive, particularly in active screening, which is in contrast with earlier reports. While CATT has been extensively evaluated and used in clinical settings, and its sensitivity has been reported to range between 68.8% and 100% [22], in this study, the test missed almost half of the cases in active screening. Previous retrospective studies also reported the sensitivity of the RDT1 to be between 82% and 99.6% [8,11]. A sensitivity of 89% was reported in a prospective study of a prototype version of the RDT1 [9], while in another trial, the sensitivity of the commercialized RDT1 was 92% [14]. This apparent discrepancy could be explained by assuming that each of the three screening tests detected cases with different serological profiles, which were only partially overlapping, as evidenced by the results shown in Fig 4. The design of the study, which included three screening tests to identify suspects during enrolment, would be responsible for the low sensitivity of an individual screening test. By contrast, earlier studies only included one, or sometimes two screening tests during enrolment, and as a result, the sensitivity of screening tests could have been significantly overestimated, since cases with serological profiles that were different from the ones identified by the particular test could have been missed. Therefore, there is the need to explore the possibility of including two or more screening tests in diagnostic algorithms, in order to increase sensitivity and accelerate interruption of disease transmission, particularly by enhancing detection of patients in early stage disease. Based on the results presented here, strategies combining RDT2 with either RDT1 or CATT in active screening, and combining RDT2 with RDT1 in passive screening, could be considered to enhance case detection. In active screening, each test detected a particularly large number of cases that were missed by the other tests, and combining several screening tests would therefore result in a stronger gain in sensitivity than in passive screening. However, operational aspects would also need to be considered, and cost-effectiveness analyses may provide helpful information to select the most appropriate strategies that would ensure optimal detection of cases. In particular, there is the need to determine whether the extra complexity of the diagnostic algorithm and workload that would result from performing two or more screening tests and having more serological suspects to test by microscopy would cause a significant reduction in the number of people screened by a mobile team in a day, and balance it against the gain in detection of a larger proportion of cases among the people screened. Performing several screening tests would also be a logistical challenge in terms of transportation and storage of tests. Some patients could also refuse to have two or more tests performed on them, an unlikely possibility since blood is taken from the same finger prick. If only one screening test had to be used, the results presented here support using RDT2 in order to enhance case detection, as it was more sensitive than RDT1 and CATT, in both active and passive screening settings. RDT2 was also the most sensitive test in both early and late stage patients, which indicates that the test is able to detect patients with various clinical profiles. Maximizing sensitivity would be a sensible strategy in a disease elimination context, but the marginally lower specificity of RDT2 would also need to be considered, as it would result in an increase in workload to confirm suspects, decrease in confidence in test results and would also have a negative impact on patients, since a larger number of suspects would need to undergo confirmatory testing, which often requires travelling long distances. Such limitations will become increasingly relevant as progress is being made towards elimination of the disease, since the positive predictive value of screening tests will decline along with the disease prevalence.

Alternatively, investing in the development of a new screening test that would be more sensitive than the tests that were evaluated here, and which would include multiple antigens, could be considered. Such a test might be developed by combining three or more antigens, which could include those in the RDT2, as well as other promising candidates identified in previous studies [23–28]. Other RDTs being developed using recombinant antigens will also need to be considered once they are available and their performance has been evaluated [25]. With the increasing prospects of new, safer treatments for g-HAT that would be effective for both stages of the disease [29,30], a test with high sensitivity and specificity could make a “test and treat” approach possible, without requiring any parasitological confirmation.

A number of hypotheses could be formulated to try and explain the low sensitivity of individual screening tests observed in this study, which would require further investigations. African trypanosomes are notorious for having evolved a mechanism of escaping the host immune system by regularly changing the variant surface glycoprotein (VSG) that composes their cell coat, using a large repertoire of dedicated genes [31,32]. It is therefore likely that HAT patients who have been infected recently could have raised an immune response to only a limited number of VSG antigens, while patients who are in a more advanced disease stage could harbour antibodies against a larger panel of VSGs. This could explain why screening tests that include some specific VSGs, such as the three tests evaluated here, would detect different HAT cases, and why screening tests were more sensitive in late than in early disease stage patients. Similarly, this would provide an explanation for the lower sensitivity that was observed in active screening, since the disease is generally less advanced in most cases among the people screened. Alternatively, the difference in sensitivity between early and late-stage patients could be due to higher antibody titres in the latter because of a longer period of exposure to parasite antigens, and hence stronger immune response. This explanation would better support the finding that the sensitivity of an invariant antigen like ISG65, which is expressed throughout the infection, was higher in late-stage patients. These hypotheses could be tested using animal models infected with *T*.*b*. *brucei* [32]. Some patients could have also been infected with trypanosome strains lacking the genes encoding the VSG antigens present in these screening tests. In particular, deletions of the gene encoding VSG LiTat 1.3 have been reported in some *T*.*b*. *gambiense* isolates from Cameroon [33], and such deletions could be among the factors responsible for the low sensitivity of CATT and RDT1. Although there is currently no evidence to directly support this hypothesis, it is also conceivable that these deletions could have become increasingly frequent due to the selection pressure applied by the extensive use of CATT in HAT-endemic populations. This phenomenon could have remained unnoticed, since most studies conducted until recently only included CATT during enrolment. Finally, it cannot be excluded that some HAT cases could have corresponded to false positive parasitological test results, which would have been negative with screening tests. It is likely that several of the hypotheses described here could partially explain the observed low sensitivity of screening tests that was found in this study. Other studies comparing the performance of different screening tests in various settings will hopefully help clarify this point.

The fact that RDT1 detected 49 HAT cases that were missed by CATT (Fig 4) could be explained by the presence in RDT1 of the VSG LiTat 1.5 antigen, which is not included in CATT, and also possibly by differences in test formats. On the other hand, it is noteworthy that CATT also detected 58 HAT cases that were missed by RDT1, yet RDT1 contains VSG LiTat 1.3, the antigen that is predominantly expressed by the fixed trypanosomes in the CATT test. This could be due to the nature of the CATT reagents, which in addition to VSG LiTat 1.3, would include other trypanosome antigens that could react with corresponding antibodies in the blood of HAT patients. Another explanation might be the difference in test formats, which may be associated with different binding or exposure characteristics of antigens and epitopes. While in the RDT, antigens are printed on a nitrocellulose membrane, CATT is performed by mixing a suspension containing fixed parasites with the test sample on a plasticised card. In addition, although the exact composition of the RDT buffer is unknown, it is likely to be different from the CATT buffer (phosphate buffered saline, pH 7.2 with 0.1% sodium azide), which could have an impact on antigenic binding.

In an earlier prospective study that was conducted in the DRC to evaluate the performance of RDT1, the VSG LiTat 1.5 antigen was more sensitive than the VSG LiTat 1.3 antigen (83.6% [CI: 76.3%; 89.0%] and 76.0% [CI: 68.0%; 82.5%], respectively) [14], which is in contrast to what was found in the present study. In another multi-country study that evaluated the performance of the prototype RDT1, identical sensitivity values were reported for each antigen (85.9% [CI: 79.4%; 90.6%]) [9]. While these differences may be due to slightly different study designs, they do not appear to be statistically significant, and would therefore tend to support the view that both antigens contribute equally to the sensitivity of RDT1.

While the three screening tests were highly specific in active screening, they were significantly less specific in passive screening. This difference might be due to serological differences between the two populations, with the population presenting to fixed health facilities being more likely to be infected with other pathogens that could trigger immune responses cross-reacting with the tests. Alternatively, this difference might be explained by the relatively low sensitivity of routine parasitological methods [34]. Indeed, since the HAT prevalence was higher in passive than in active screening, this population was also more likely to have included HAT patients who could have been found positive by screening tests but missed by parasitology, which would have resulted in an underestimate of the specificity of screening tests. The difference in specificity between active and passive screening could thus be an artefact related to the imperfect parasitological reference standard, rather than reflect a real difference in test specificity.

RDT1 and CATT were previously evaluated in another prospective study that was conducted in the DRC [14], which reported that the sensitivity of RDT1 (92.0% [CI: 86.1%-95.5%]) was significantly higher than that of CATT (69.1% [CI: 60.7%-76.4%]). Surprisingly, there was no evidence of any difference in sensitivity between RDT1 and CATT in the present study. The reasons for this discrepancy are unclear, and several hypotheses could be drawn. First, although the studies shared some of the sites, it is possible that the two study populations may have had significantly distinct serological profiles, resulting in different degrees of overlap between the tests. According to this hypothesis, the degree of overlap between screening tests should not be viewed as constant and specific to the tests, but instead, considered as a dynamic phenomenon that may exhibit significant variability in time and in space, depending on the population that is sampled and the underlying immune responses of individual patients. Although this hypothesis seems rather unlikely since the studies were conducted in similar populations, it would be useful to conduct additional studies to establish the reproducibility of such differences. In spite of efforts to ensure compliance with the study protocol and procedures through training, supervision and monitoring, it is still possible that some of the sites could have performed less well, which could have had an impact on study results. Alternatively, differences between these studies could be due to operational or logistical factors causing some of the tests to have a lower performance than expected. While this seems unlikely, subtle changes during the production of antigens or other components of one of the tests could have occurred and gone undetected, resulting in the lower sensitivity of some test batches. No failure to follow storage procedures was observed during the study, screening tests were used according to manufacturers’ instructions and staff performing the tests ensured that positive and negative controls (for CATT) as well as procedural controls (for RDTs) reacted according to instructions. Yet it is possible that some tests could have deteriorated within the limits of the controls, thereby affecting performance.

The mAECT-bc method [17] may be considered as a replacement of mAECT-wb, which is routinely used in the DRC and other endemic countries. Although based on a subset of participants, the data presented here are in agreement with earlier results showing a significant increase in sensitivity using mAECT-bc. In a first study that was conducted in Guinea, the sensitivity of mAECT-bc was 96.5%, while the sensitivity of mAECT-wb was 78.9% [17]. Another study that was conducted in DRC reported a somehow smaller difference in sensitivity between these two methods (90.9% and 80.4%, respectively) [34]. The lower sensitivity values that were found here (72.6% and 51.6%, respectively) could be explained by the fact that the mAECT methods were only performed on a subset of participants who had been negative with other parasitological methods, and who were therefore likely to include cases with a lower parasitaemia than the other cases that were enrolled in the study. This selection bias could also explain why the difference in sensitivity was higher than in previous reports, since patients with a low parasitaemia could have provided a better dynamic range to evaluate subtle differences in sensitivity. While the difference in sensitivity could be an overestimate of the true difference that would be observed in an unbiased population, implementing the mAECT-bc protocol could be considered to enhance case finding, for a minimal additional workload. Since there was a high degree of overlap between the mAECT-wb and mAECT-bc results, performing both methods may not be justified, as it would increase costs without resulting in any significant increase in sensitivity. Although introducing mAECT-bc would require specific training to prepare buffy coat samples, it did not present a particular challenge during this study, and therefore, implementing it at other sites that are already equipped to perform mAECT-wb should be relatively straightforward.

While mAECT-bc has been shown to be more sensitive than mAECT-wb, mAECT-wb is known to be more sensitive than CTC [6,34]. Thus, the observation that the positivity rates of the screening tests were highest in cases found positive by mAECT-bc and lowest in cases that were positive by CTC could suggest that screening tests would be more sensitive in low-parasitaemia than in high-parasitaemia cases. Although this would need to be further investigated, it would be in agreement with the assumption that patients with a low parasitaemia would have a stronger immune response, which would facilitate their identification using antibody-detection screening tests. Conversely, patients unable to mount a strong immune response against trypanosomes and therefore more likely to have a high parasitaemia could be more difficult to identify using these screening tests.

This study confronted a number of challenges, which could have somehow impacted the quality of the results. Although study sites were carefully selected based on the available epidemiological data, the HAT prevalence was generally low, making it necessary to enroll patients at 15 different sites. This presented a significant challenge to the study team in terms of coordination, in particular when considering that most of the sites are located in remote, rural areas that were difficult to access. In addition, there was significant turn-over of personnel at some of the sites, requiring additional training. Enrolment was also interrupted at some sites due to stock-outs of supplies, such as mAECT kits. While the study was blinded, it is possible that technicians performing the tests could have been aware of the clinical status of some participants. This is probably more likely to be true in passive screening, since the number of patients presenting daily to health facilities was sometimes very low, making blinding more difficult. Although it is hypothetical, this imperfect blinding could be one of the factors leading to the high degree of overlap of true positive results, and to a lesser extent of false positive results, between the tests that was found in passive screening.

The results presented here have confirmed that the RDT2 would be a useful test for both active and passive screening, either as a single test or in combination with other screening tests. Since it is produced using recombinant antigens exclusively, it will also be easier and safer to manufacture than screening tests that are made with native antigens. The RDT2 is thus a welcome addition to the set of tools that are currently available to control and eventually eliminate HAT.

## Supporting information

S1 Fig Venn diagrams showing the number of true positive results obtained with the RDT2, RDT1 and CATT tests among early-stage (A) and late-stage (B) cases. For the sake of simplicity, only results obtained by the first reader are shown. The total number of true positives does not equal the total number of cases enrolled in the study (N = 260), as the first reader missed two cases in active screening.
(TIF)

S1 Table STARD checklist. (DOCX)

S2 Table Results of parasitological tests performed on HAT cases and positivity of screening tests in cases that were positive with specific parasitological tests. CTC: capillary tube centrifugation; mAECT-wb: mini anion exchange centrifugation technique on whole blood; mAECT-bc: mini anion exchange centrifugation technique on buffy coat.
(DOCX)

S3 Table Malaria RDT results obtained in HAT cases and serological suspects. Malaria prevalence values correspond to the percentage of positive malaria RDT results obtained among participants that were tested with a malaria RDT. For the sake of simplicity, only results obtained by the first reader are shown.
(DOCX)

S4 Table Sensitivity of HAT screening tests in malaria RDT positive and negative participants in passive screening. For the sake of simplicity, only results obtained by the first reader are shown.
(DOCX)

S1 Data Study database. (CSV)
