# Supplementary figures and images for: Prospective evaluation of a rapid diagnostic test for Trypanosoma brucei gambiense infection developed using recombinant antigens
Source: PLoS Negl Trop Dis. 2018 Mar 28;12(3):e0006386. doi: 10.1371/journal.pntd.0006386 (PMC5898764; doi:10.1371/journal.pntd.0006386)

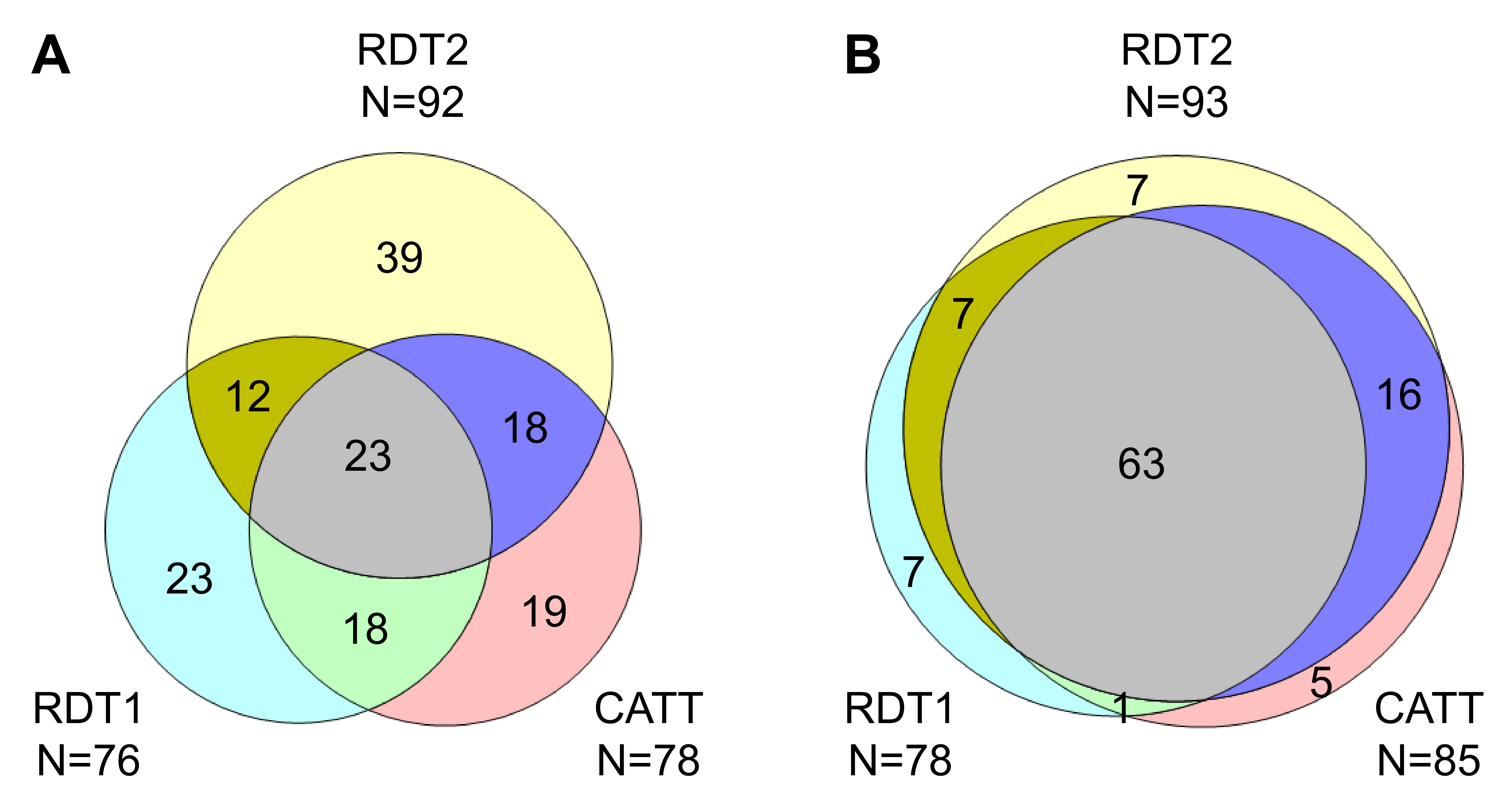

Supplement: S1 Fig — For the sake of simplicity, only results obtained by the first reader are shown. The total number of true positives does not equal the total number of cases enrolled in the study (N = 260), as the first reader missed two cases in active screening. (TIF) [file pntd.0006386.s001.tif]
