# Supplementary material for: Prospective evaluation of a rapid diagnostic test for Trypanosoma brucei gambiense infection developed using recombinant antigens
Source: PLoS Negl Trop Dis. 2018 Mar 28;12(3):e0006386. doi: 10.1371/journal.pntd.0006386 (PMC5898764; doi:10.1371/journal.pntd.0006386)
Supplement: S1 Table — (DOCX) [file pntd.0006386.s002.docx]

**S1 Table. STARD checklist.**

| **Section & Topic** | **No** | **Item** | **Reported in manuscript section (paragraph)** |
| --- | --- | --- | --- |
|  |  |  |  |
| **TITLE OR ABSTRACT** |  |  |  |
|  | **1** | Identification as a study of diagnostic accuracy using at least one measure of accuracy  (such as sensitivity, specificity, predictive values, or AUC) | Abstract (paragraph 2) |
| **ABSTRACT** |  |  |  |
|  | **2** | Structured summary of study design, methods, results, and conclusions  (for specific guidance, see STARD for Abstracts) | Abstract |
| **INTRODUCTION** |  |  |  |
|  | **3** | Scientific and clinical background, including the intended use and clinical role of the index test | Introduction (paragraphs 1-3) |
|  | **4** | Study objectives and hypotheses | Introduction (paragraph 4) |
| **METHODS** |  |  |  |
| *Study design* | **5** | Whether data collection was planned before the index test and reference standard  were performed (prospective study) or after (retrospective study) | Introduction (last paragraph) / Methods (paragraph 1) |
| *Participants* | **6** | Eligibility criteria | Methods (paragraph 1) |
|  | **7** | On what basis potentially eligible participants were identified  (such as symptoms, results from previous tests, inclusion in registry) | Methods (paragraph 1) |
|  | **8** | Where and when potentially eligible participants were identified (setting, location and dates) | Methods (paragraph 1) |
|  | **9** | Whether participants formed a consecutive, random or convenience series | Methods (paragraph 1) |
| *Test methods* | **10a** | Index test, in sufficient detail to allow replication | Methods (paragraph 2) |
|  | **10b** | Reference standard, in sufficient detail to allow replication | Methods (paragraph 1) |
|  | **11** | Rationale for choosing the reference standard (if alternatives exist) | NA |
|  | **12a** | Definition of and rationale for test positivity cut-offs or result categories of the index test, distinguishing pre-specified from exploratory | NA |
|  | **12b** | Definition of and rationale for test positivity cut-offs or result categories  of the reference standard, distinguishing pre-specified from exploratory | NA |
|  | **13a** | Whether clinical information and reference standard results were available to the performers/readers of the index test | Methods (paragraph 5) |
|  | **13b** | Whether clinical information and index test results were available  to the assessors of the reference standard | Methods (paragraph 4) |
| *Analysis* | **14** | Methods for estimating or comparing measures of diagnostic accuracy | Methods (paragraphs 6 and 7) |
|  | **15** | How indeterminate index test or reference standard results were handled | Methods (paragraphs 2 and 4) |
|  | **16** | How missing data on the index test and reference standard were handled | Methods (paragraph 4) |
|  | **17** | Any analyses of variability in diagnostic accuracy, distinguishing pre-specified from exploratory | Discussion (paragraphs 1, 3, 4 and 6) |
|  | **18** | Intended sample size and how it was determined | Methods (paragraph 8) |
| **RESULTS** |  |  |  |
| *Participants* | **19** | Flow of participants, using a diagram | Methods (paragraph 1) (no diagram) |
|  | **20** | Baseline demographic and clinical characteristics of participants | NA |
|  | **21a** | Distribution of severity of disease in those with the target condition | NA |
|  | **21b** | Distribution of alternative diagnoses in those without the target condition | NA |
|  | **22** | Time interval and any clinical interventions between index test and reference standard | Methods (paragraph 4) |
| *Test results* | **23** | Cross tabulation of the index test results (or their distribution)  by the results of the reference standard | NA. Results were calculated using a bootstrapping resampling approach (Methods, paragraph 6). |
|  | **24** | Estimates of diagnostic accuracy and their precision (such as 95% confidence intervals) | Results, Fig 1. |
|  | **25** | Any adverse events from performing the index test or the reference standard | NA |
| **DISCUSSION** |  |  |  |
|  | **26** | Study limitations, including sources of potential bias, statistical uncertainty, and generalisability | Discussion (paragraphs 3, 6, 8 and 9). |
|  | **27** | Implications for practice, including the intended use and clinical role of the index test | Discussion (paragraphs 1 and 10). |
| **OTHER INFORMATION** |  |  |  |
|  | **28** | Registration number and name of registry | NA |
|  | **29** | Where the full study protocol can be accessed | NA |
|  | **30** | Sources of funding and other support; role of funders | Financial disclosure |
|  |  |  |  |
