# Supplementary material for: Prospective evaluation of a rapid diagnostic test for Trypanosoma brucei gambiense infection developed using recombinant antigens
Source: PLoS Negl Trop Dis. 2018 Mar 28;12(3):e0006386. doi: 10.1371/journal.pntd.0006386 (PMC5898764; doi:10.1371/journal.pntd.0006386)
Supplement: S2 Table — CTC: capillary tube centrifugation; mAECT-wb: mini anion exchange centrifugation technique on whole blood; mAECT-bc: mini anion exchange centrifugation technique on buffy coat. (DOCX) [file pntd.0006386.s003.docx]

|  |  | **Parasitological test results** | | | **RDT1** | | **RDT2** | | **CATT** | |
| --- | --- | --- | --- | --- | --- | --- | --- | --- | --- | --- |
| **Parasitological test** | **Screening method** | **HAT cases tested** | **Negative** | **Positive** | **Positive^1^** | **Positivity (%)**  **(95% CI)^2^** | **Positive^1^** | **Positivity (%)**  **(95% CI)^2^** | **Positive^1^** | **Positivity (%)**  **(95% CI)^2^** |
| Lymph node aspirate | Both active and passive | 80 | 22 | 58 | 39 | 67.2 (57.0;77.5) | 42 | 72.4 (62.6;82.2) | 40 | 69.0 (58.8;79.1) |
|  | Active | 37 | 5 | 32 | 16 | 50.0 (33.9;66.1) | 16 | 50.0 (33.9;66.1) | 15 | 46.9 (30.8;63.0) |
|  | Passive | 43 | 17 | 26 | 23 | 88.5 (78.9;98.0) | 26 | 100.0 (100.0;100.0) | 25 | 96.2 (90.4;100.0) |
| CTC | Both active and passive | 214 | 110 | 104 | 57 | 54.8 (48.1;61.5) | 63 | 60.6 (54.0;67.1) | 60 | 57.7 (51.1;64.3) |
|  | Active | 111 | 40 | 71 | 37 | 52.1 (42.8;61.4) | 35 | 49.3 (40.0;58.6) | 41 | 57.7 (48.6;66.9) |
|  | Passive | 103 | 70 | 33 | 20 | 60.6 (51.2;70.0) | 28 | 84.8 (77.9;91.8) | 19 | 57.6 (48.0;67.1) |
| mAECT-wb | Both active and passive | 144 | 60 | 84 | 52 | 61.9 (54.0;69.8) | 72 | 85.7 (80.0;91.4) | 49 | 58.3 (50.3;66.4) |
|  | Active | 50 | 24 | 26 | 9 | 34.6 (21.4;47.8) | 18 | 69.2 (56.4;82.0) | 8 | 30.8 (18.0;43.6) |
|  | Passive | 94 | 36 | 58 | 43 | 74.1 (65.3;83.0) | 54 | 93.1 (88.0;98.2) | 41 | 70.7 (61.5;79.9) |
| mAECT-bc | Both active and passive | 130 | 36 | 94 | 67 | 71.3 (63.5;79.1) | 82 | 87.2 (81.5;93.0) | 71 | 75.5 (68.1;82.9) |
|  | Active | 39 | 24 | 15 | 10 | 66.7 (51.9;81.5) | 10 | 66.7 (51.9;81.5) | 13 | 86.7 (76.0;97.3) |
|  | Passive | 91 | 12 | 79 | 57 | 72.2 (62.9;81.4) | 72 | 91.1 (85.3;97.0) | 58 | 73.4 (64.3;82.5) |
| Lumbar puncture | Both active and passive | 258 | 195 | 63 | 50 | 79.4 (74.4;84.3) | 61 | 96.8 (94.7;99.0) | 57 | 90.5 (86.9;94.1) |
|  | Active | 137 | 132 | 5 | 3 | 60.0 (51.8;68.2) | 4 | 80.0 (73.3;86.7) | 4 | 80.0 (73.3;86.7) |
|  | Passive | 121 | 63 | 58 | 47 | 81.0 (74.0;88.0) | 57 | 98.3 (96.0;100.0) | 53 | 91.4 (86.4;96.4) |

^1^ Number of positive results obtained among cases that were positive with the parasitological test. For the sake of simplicity, only results obtained by the first reader are shown.

^2^ Percentage calculated as the number of positive results obtained among cases that were positive with the parasitological test divided by the number of cases that were positive with the parasitological test.
