# Supplementary material for: Prospective evaluation of a rapid diagnostic test for Trypanosoma brucei gambiense infection developed using recombinant antigens
Source: PLoS Negl Trop Dis. 2018 Mar 28;12(3):e0006386. doi: 10.1371/journal.pntd.0006386 (PMC5898764; doi:10.1371/journal.pntd.0006386)
Supplement: S3 Table — Malaria prevalence values correspond to the percentage of positive malaria RDT results obtained among participants that were tested with a malaria RDT. For the sake of simplicity, only results obtained by the first reader are shown. (DOCX) [file pntd.0006386.s004.docx]

|  |  | **Malaria RDT** | | |  |
| --- | --- | --- | --- | --- | --- |
| **Participant group** | **Screening method** | **Negative** | **Positive** | **Not done** | **Malaria prevalence (%) (95% CI)** |
| HAT cases | Both active and passive | 149 | 80 | 31 | 34.9 (28.8;41.1) |
|  | Active | 89 | 25 | 24 | 21.9 (14.3;29.5) |
|  | Passive | 60 | 55 | 7 | 47.8 (38.7;57.0) |
| RDT1 positives | Both active and passive | 433 | 216 | 132 | 33.3 (29.7;36.9) |
|  | Active | 183 | 54 | 107 | 22.8 (17.4;28.1) |
|  | Passive | 250 | 162 | 25 | 39.3 (34.6;44.0) |
| RDT2 positives | Both active and passive | 647 | 441 | 203 | 40.5 (37.6;43.5) |
|  | Active | 244 | 102 | 167 | 29.5 (24.7;34.3) |
|  | Passive | 403 | 339 | 36 | 45.7 (42.1;49.3) |
| CATT positives | Both active and passive | 340 | 227 | 75 | 40.0 (36.0;44.1) |
|  | Active | 154 | 69 | 67 | 30.9 (24.9;37.0) |
|  | Passive | 186 | 158 | 8 | 45.9 (40.7;51.2) |
