# Supplementary material for: Prospective evaluation of a rapid diagnostic test for Trypanosoma brucei gambiense infection developed using recombinant antigens
Source: PLoS Negl Trop Dis. 2018 Mar 28;12(3):e0006386. doi: 10.1371/journal.pntd.0006386 (PMC5898764; doi:10.1371/journal.pntd.0006386)
Supplement: S4 Table — For the sake of simplicity, only results obtained by the first reader are shown. (DOCX) [file pntd.0006386.s005.docx]

| **Malaria RDT result** | **HAT screening test** | **Sensitivity (%) (95% CI)** |
| --- | --- | --- |
| Positive (n=55) | RDT2 | 89.2 (80.4;96.3) |
|  | RDT1 | 59.4 (46.5;72.5) |
|  | CATT | 72.4 (60.0;83.8) |
| Negative (n=60) | RDT2 | 91.6 (84.5;98.2) |
|  | RDT1 | 80.7 (69.8;90.3) |
|  | CATT | 81.4 (71.2;91.1) |
